# Pedal to the Metal: Nuclear Splicing Bodies Turbo-Charge VSG mRNA Production in African Trypanosomes

**DOI:** 10.3389/fcell.2022.876701

**Published:** 2022-04-20

**Authors:** James Budzak, Gloria Rudenko

**Affiliations:** Department of Life Sciences, Sir Alexander Fleming Building, Imperial College London, London, United Kingdom

**Keywords:** variant surface glycoprotein, antigenic variation, nuclear bodies, trans-splicing, *Trypanosoma brucei*, nuclear architecture

## Abstract

The African trypanosome *Trypanosoma brucei* is a parasite of the mammalian bloodstream and tissues, where an antigenically variable Variant Surface Glycoprotein (VSG) coat protects it from immune attack. This dense layer comprised of ∼10^7^ VSG proteins, makes VSG by far the most abundant mRNA (7–10% total) and protein (∼10% total) in the bloodstream form trypanosome. How can such prodigious amounts of VSG be produced from a single VSG gene? Extremely high levels of RNA polymerase I (Pol I) transcription of the active VSG provide part of the explanation. However, recent discoveries highlight the role of pre-mRNA processing, both in maintaining high levels of VSG transcription, as well as its monoallelic expression. Trypanosome mRNAs are matured through trans-splicing a spliced leader (SL) RNA to the 5’ end of precursor transcripts, meaning abundant SL RNA is required throughout the nucleus. However, requirement for SL RNA in the vicinity of the active VSG gene is so intense, that the cell reconfigures its chromatin architecture to facilitate interaction between the SL RNA genes and the active VSG. This presumably ensures that sufficient localised SL RNA is available, and not limiting for VSG mRNA expression. Recently, novel nuclear splicing bodies which appear to provide essential trans-splicing components, have been identified associating with the active VSG. These observations highlight the underappreciated role of pre-mRNA processing in modulating gene expression in trypanosomes. Dissecting the function of these nuclear RNA processing bodies should help us elucidate the mechanisms of both VSG expression and monoallelic exclusion in *T. brucei*.

## Introduction

Nuclear bodies are increasingly being shown to be essential for the regulation and compartmentalisation of gene expression (Shin et al., 2018). These membraneless nuclear condensates facilitate vital functions in different organisms and cell types (Banani et al., 2017). They self-assemble through phase separation, functioning as “hot-spots” for specific nuclear processes. Through sequestering and concentrating proteins and RNA, they can increase reaction kinetics, and can co-ordinate inter-chromosomal interactions (Shin and Brangwynne, 2017). Cajal bodies for example, are specialised in the modification of small nuclear RNAs (snRNAs), and the assembly of splicing small nuclear ribonucleoproteins (snRNPs). The nucleolus is specialised in RNA polymerase I (Pol I) transcription of the ribosomal DNA (rDNA), processing and modification of rRNA, and ribosome assembly.

**FIGURE 1:**
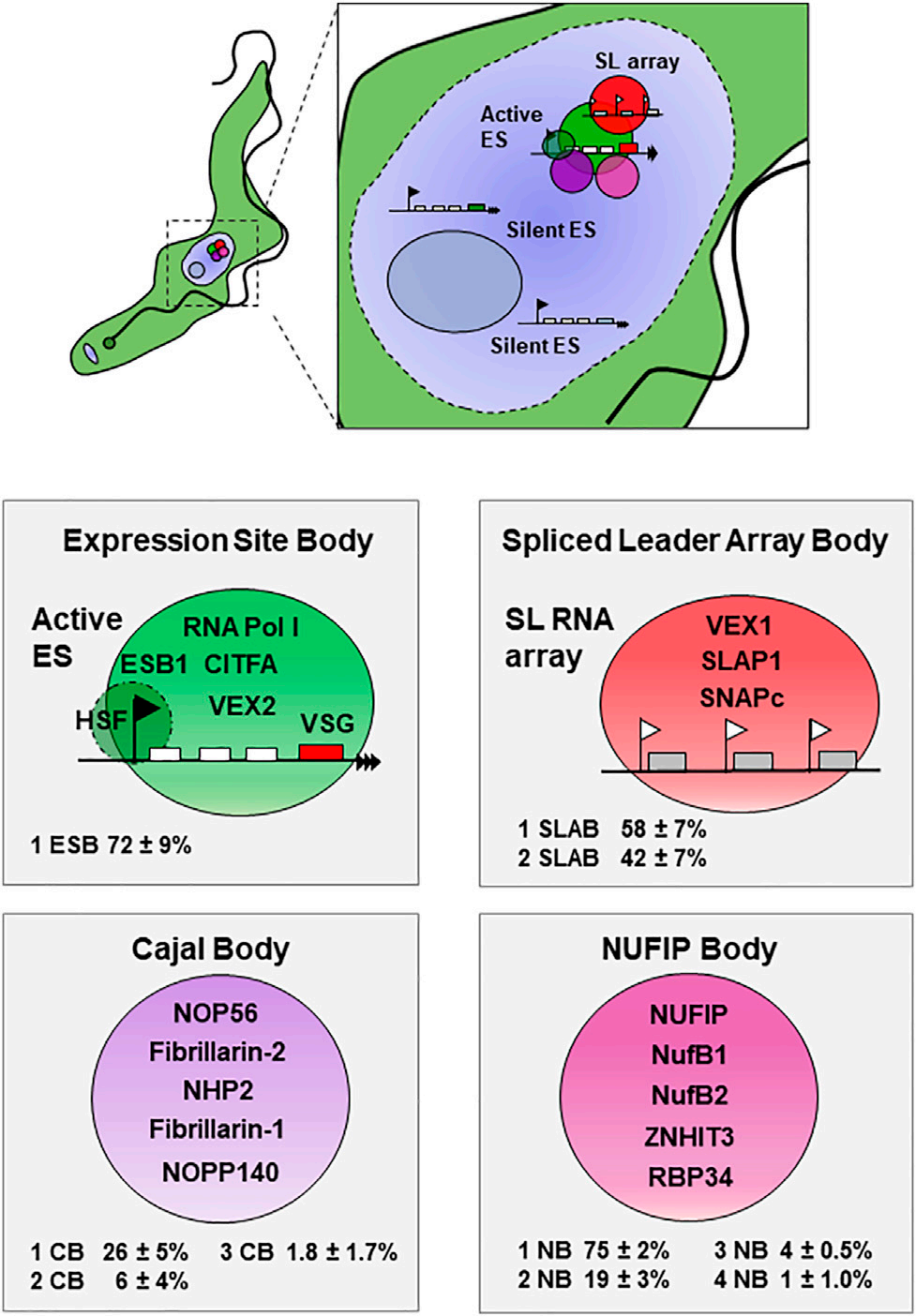
An assembly of nuclear bodies congregate at the active VSG expression site At the top a bloodstream form (BF) trypanosome is shown with the nucleus (dashed circle) magnified. This contains an assembly of nuclear bodies (coloured circles) at the active VSG expression site (ES), as well as a nucleolus (blue circle). The active ES has a Pol I promoter indicated with a black flag, various Expression site associated genes (ESAGs) with white boxes, and the VSG with a red box (not drawn to scale). The active ES associates with at least one of the spliced leader (SL) RNA gene arrays, shown with individual SL RNA genes (grey boxes) transcribed from Pol II promoters (white flags). Nuclear bodies are shown below as large coloured circles with selected associated protein components indicated. The Expression site body (ESB) (large green circle) at the active ES is associated with a highly SUMO-ylated focus (HSF) (dark green circle). The Spliced Leader Array Body (SLAB) (red circle) associates with the SL RNA gene arrays, of which at least one is associated with the active ES. Two additional nuclear splicing bodies associating with the active ES include the Cajal Body (violet circle) and a novel NUFIP body (purple circle). These nuclear bodies (with the exception of the ESB) are also found in procyclic form *T. brucei.* The percentage of BF *T. brucei* cells in G_1_ containing one or more of these nuclear bodies is shown in the respective panels [data from (Budzak et al., 2022)].

The African sleeping sickness parasite *Trypanosoma brucei* contains a Pol I-enriched nucleolus. However, it is unique among eukaryotes in using Pol I to transcribe some protein coding genes, including *VSG*. *T. brucei* contains thousands of *VSG* genes, of which one is transcribed at a time from one of ∼15 expression site (ES) transcription units (Hertz-Fowler et al., 2008; Cross et al., 2014). The active ES is located within an extra-nucleolar Pol I body called the Expression Site Body (ESB) (Navarro and Gull, 2001) (Figure 1). There has been intense interest in factors facilitating high levels of ES transcription, and numerous chromatin proteins have been identified (Pena et al., 2017). However, we are increasingly realising the importance of pre-mRNA processing both in maintaining high levels of *VSG* expression, as well as its monoallelic control. The two processes of transcription and splicing appear to be interconnected at the active ES, whereby blocking splicing results in strongly reduced processive transcription (Budzak et al., 2022). In this review, we discuss the role of nuclear bodies in facilitating high *VSG* expression levels.

### The ESB Facilitates Extremely High Rates of Monoallelic Transcription of VSG

**FIGURE 2:**
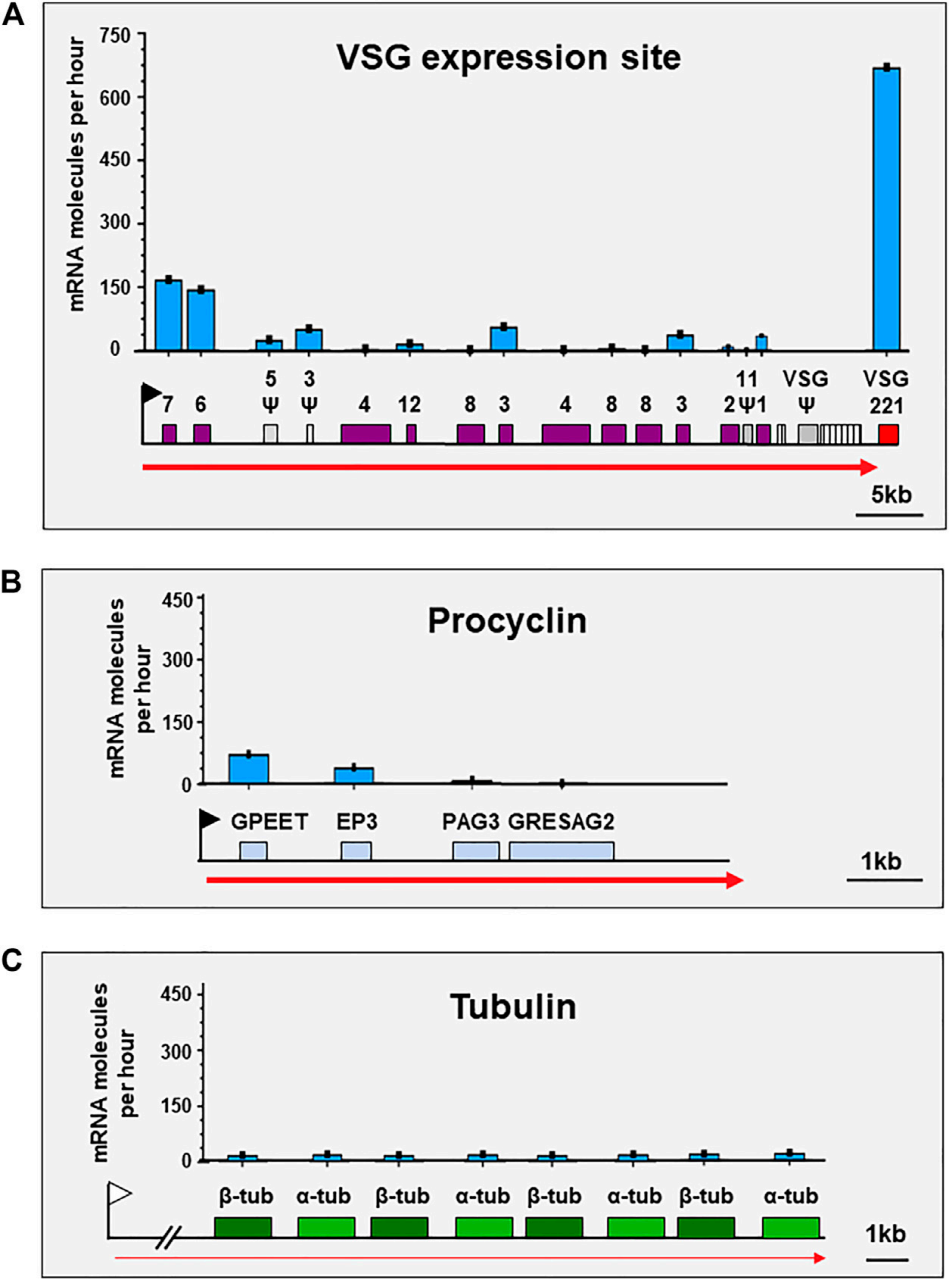
VSG is the mRNA generated at the highest rate in bloodstream form *T. brucei*. Schematics show the estimated number of mRNA molecules produced per hour from genes in different genomic loci in bloodstream form *Trypanosoma brucei*. **(A)** Schematic of an active *VSG* expression site containing the most abundant mRNA expressed in bloodstream form *T. brucei*; VSG. The Pol I ES promoter is indicated with a black flag, and high amounts of Pol I transcription with a thick red arrow. Expression Site Associated Genes (*ESAG*s) and *VSG221* are indicated with coloured boxes, pseudogenes with *Ψ* (grey boxes), and 70 bp repeats with striped boxes. **(B)** Schematic showing one of the four procyclin loci, which contain the most abundant mRNAs expressed in procyclic form *T. brucei*. The Pol I promoter is indicated with a black flag, high levels of Pol I transcription with a thick red arrow, and relevant genes with coloured boxes. **(C)** Schematic of the tubulin locus, which contains the most abundant mRNAs transcribed by Pol II in *T. brucei*. The upstream Pol II promoter is indicated with a white flag, and low levels of Pol II transcription with a thin red arrow. Three pairs of alternating α-tubulin and *ß*-tubulin genes (out of a total of eight per locus) are indicated with coloured boxes. For all graphs, the *y*-axes are the same scale as in panel **(A)**. Values for mRNA molecules generated per hour were derived from Supplementary Tables 1, 2 in Budzak et al. (2022).

The ESB is the first nuclear body shown to be key for monoallelic VSG expression (Navarro and Gull, 2001). A stringent restriction ensures that maximally one ESB is stably present within the bloodstream form (BF) trypanosome. Forced activation of a second ES, results in two ESs sharing the same ESB (Chaves et al., 1999; Budzak et al., 2019). VSG is essential for BF *T. brucei*, and extraordinarily high levels of continuous *VSG* mRNA production are required for proliferation (Sheader et al., 2005; Ridewood et al., 2017). *VSG* is by far the most abundant mRNA (∼7–10% total) in BF *T. brucei*. An estimated 666.7 *VSG* mRNA molecules are generated per hour from a single *VSG* gene, compared with 1.3 mRNA molecules per hour from a typical Pol II transcribed gene (Budzak et al., 2022) (Figure 2). This staggering 512-fold higher rate of mRNA production, enables generation of the vast amounts of Variant Surface Glycoprotein (VSG) (10^7^ molecules, ∼10% total protein) necessary to form a fully protective surface coat (Bartossek et al., 2017; Maudlin et al., 2021). The ESB can therefore be considered a specialised transcription factory allowing enormous amounts of *VSG* pre-mRNA to be transcribed.

Such an extreme requirement for mRNA production does not occur in many cell types. Something similar is seen in antibody secreting plasma cells. Antibody production in B cells can reach 10^8^ immunoglobulin molecules per hour (Hibi and Dosch, 1986), with an extraordinary 70% of the mRNA transcriptome comprised solely of immunoglobulin (IgG) mRNAs (Shi et al., 2015). To achieve this, transcription of immunoglobulin heavy chain genes is boosted by super-enhancers (Le Noir et al., 2017). Genes present on different chromosomes colocalise in transcription factories located in activating regions at the nuclear periphery, thereby facilitating enhancer interactions (Park et al., 2014).

African trypanosomes have evolved their own unusual adaptations to cope with the extreme biology of producing such large amounts of mRNA from a single *VSG* gene. One of these is the incredible stability of the *VSG* mRNA. *VSG* mRNA has one of the longest half-lives in the cell, which is partly conferred by a specific RNA binding protein (CFB2), which recognises conserved elements in the *VSG* 3′UTR (Ridewood et al., 2017; Melo do Nascimento et al., 2021). N^6^-methyladenosine modification of the *VSG* poly(A) tails has also been proposed as a mechanism for increasing *VSG* mRNA stability (Viegas et al., 2020). An additional important adaptation is the use of Pol I for ES transcription (Gunzl et al., 2003). In mammalian cells, Pol I initiates transcription at significantly higher rates than Pol II, with a reinitiation interval of one polymerase per ∼1.4 s (Dundr et al., 2002), compared with an initiation rate for Pol II which is frequently less than ∼1-2 per minute (Darzacq et al., 2007; Fuchs et al., 2014). In eukaryotes Pol I normally exclusively transcribes rDNA, as it generates uncapped, and therefore untranslateable transcripts (Grummt and Skinner, 1985). In trypanosomes however, as *trans*-splicing adds capped Pol II derived spliced leader (SL) RNA to the 5′ end of mRNA precursors (Gunzl, 2010), Pol I derived mRNAs are rendered translateable. In *T. brucei* it is estimated that a Pol I transcribed gene is expressed at a more than ∼10-fold higher rate than one transcribed by Pol II (Biebinger et al., 1996). Trypanosomes therefore appear to have co-opted the highest initiating polymerase in the cell (Pol I), to maximise transcription of *VSG*.

Both Pol I bodies, the ESB and the nucleolus, are enriched for Pol I transcription factors including CITFAs and the architectural chromatin protein TDP1 (Narayanan and Rudenko, 2013; Nguyen et al., 2014). However, the ESB also contains components not found in the nucleolus including VEX2, which contains homology to the RNA helicase UPF1. Interestingly, depletion of VEX2 results in upregulation of *ESAG*s from the active ES, suggesting it may play a role in suppressing excessive *ESAG* mRNA production (Faria et al., 2019). In addition, the first ESB-specific protein (ESB1) has now been discovered, which could be involved in protein ubiquitinylation (Escobar et al., 2021). Both VEX2 and ESB1 are important for monoallelic VSG expression, as their perturbation results in upregulation of silent ESs. A highly SUMOylated focus is also observed at the active ES, but not the nucleolus, and could play a role in ESB stabilisation (Lopez-Farfan et al., 2014).

The ESB appears to be a specialised Pol I factory, facilitating extremely high levels of transcription, and monoallelic expression of VSG. However, we are increasingly realising the importance of highly efficient pre-mRNA processing in both of these processes. ES precursor transcripts must be rapidly *trans-*spliced and polyadenylated, and blocking *trans*-splicing using chemical inhibitors or anti-U2 Morpholinos results in a radical reduction in processive ES transcription (Budzak et al., 2022). In mammalian cells there is an extensive literature on the feedback between Pol II transcription and splicing, whereby splicing can enhance Pol II transcription elongation (Tellier et al., 2020). This can operate through interaction of the phosphorylated C-terminus of Pol II with different components of the splicing machinery (Kornblihtt et al., 2004; Maita and Nakagawa, 2020). However, in *T. brucei* this would require interaction of the splicing machinery with Pol I exclusively at the active ES and not the Pol I transcribed rDNA. However alternatively, blocking *trans*-splicing could result in unspliced ES precursor transcripts remaining associated with the extending Pol I molecules, thereby facilitating polymerase removal from the template by RNA-degradation factors. This would make processing of ES precursor transcripts through *trans*-splicing of the SL exon essential for maintaining high levels of transcription by allowing unimpeded elongation. It now appears that the sheer amount of localised SL RNA required in the vicinity of the active ES has necessitated the trypanosome to reconfigure its nucleus to accommodate this.

### The Spliced Leader Array Body Interacts With the Active VSG

*T. brucei* mRNAs are generated through coupled *trans*-splicing of a capped SL RNA exon and polyadenylation (Matthews et al., 1994; Clayton, 2019). This allows the production of translateable mRNA from precursor transcripts from the extensive polycistronic transcription units comprising most of the *T. brucei* genome (Berriman et al., 2005). The large amount of SL RNA required is generated from two arrays of over 100 SL RNA genes, each with its own Pol II promoter (Gilinger and Bellofatto, 2001). SL RNA transcription is mediated by a specialised SNAP transcription factor complex (Schimanski et al., 2005). Two additional proteins localising at the SL RNA array in both BF and procyclic form (PF) *T. brucei* are VEX1 and Spliced Leader Array Protein 1 (SLAP1), (Faria et al., 2021) (Budzak et al., 2022). VEX1 is important for maintenance of monoallelic expression of VSG, but is nonessential (Glover et al., 2016). SLAP1 is essential, and its knockdown results in decreased amounts of the SL RNA intron, as well as defective splicing (Budzak et al., 2022).

The proteins colocalising at the SL RNA array allow identification of the Spliced Leader Array Body (SLAB), of which there are one or two, in both BF and PF *T. brucei* (Faria et al., 2021; Budzak et al., 2022) (Figure 1). The SLAB associates with the active ES in BF *T. brucei*, and when cells have two SLAB, at least one is typically within 350 nm of the ESB (Glover et al., 2016; Escobar et al., 2021; Faria et al., 2021; Budzak et al., 2022). This agrees with the surprising discovery made using Hi-C chromosome conformation capture experiments, that there is a robust inter-chromosomal interaction between the SL RNA array and the active ES (Faria et al., 2021). This contact could be mediated by the VEX1-VEX2 complex acting as a bridge between the SL-RNA array and the active *VSG* ES. However, only depletion of VEX2 (and not VEX1) disrupts this interaction, (Faria et al., 2021) (Budzak et al., 2022). This could indicate additional bridging molecules facilitate interaction of these two loci.

### The Cajal and NUFIP Bodies

SL RNA *trans-*splicing in trypanosomes requires spliceosomal small nuclear ribonucleoproteins (snRNPs) (Gunzl, 2010), which are modified and assembled in Cajal bodies (Morris, 2008; Meier, 2017). Cajal bodies, while absent in many cell types, are typically found in rapidly dividing embryonic or cancer cells. Their presence and abundance is thought to be correlated to splicing rates (Young et al., 2000; Morris, 2008). They can be dispensable, as knockdown of the Cajal body scaffolding protein coilin, results in defective Cajal bodies but viable mice (Tucker et al., 2001). Cajal bodies are frequently associated with highly expressed loci (Wang et al., 2016). Their function therefore appears to be the concentration of essential splicing components, thereby catalysing processes which would otherwise be rate limiting (Sawyer et al., 2016).

The Cajal body has been elusive in *T. brucei*, as coilin, the canonical Cajal body marker, is not readily identifiable in the genome. However recently, a number of new nuclear bodies present in both BF and PF *T. brucei* were identified using the TrypTag database of *T. brucei* proteins tagged with mNeonGreen (Dean et al., 2017; Budzak et al., 2022). These include a Cajal body, containing conserved extra-nucleolar Cajal body proteins. Additionally, a novel nuclear body was identified, which was called the NUFIP body (Figure 1). This contains the highly conserved NUFIP and ZNHIT3 proteins, which mediate snRNP assembly in mammalian cells (Rothe et al., 2014; Bizarro et al., 2015). The NUFIP body appears to be important in BF *T. brucei,* as 75 ± 2% of G1 cells have one NUFIP body, with most of the rest containing two. In contrast, a Cajal body is present in only 26 ± 5% BF *T. brucei* in G1 (Budzak et al., 2022), making it unclear if it is essential. In PF *T. brucei* the relative abundance of these two nuclear bodies is shifted, with fewer G1 cells (45 ± 5.5%) containing minimally one NUFIP body, and more (36 ± 3.5%) containing at least one Cajal body.

Similar to the SLAB, when one or more NUFIP or Cajal bodies are visible in BF *T. brucei*, minimally one of these bodies is near the active ES (Figure 1) (Budzak et al., 2022). Another feature which is similar to the SLAB, is that the nuclear positioning of the NUFIP body is determined by ES activity, as when cells switch between ESs, these bodies interact with the newly activated ES (Faria et al., 2021; Budzak et al., 2022). Despite the importance of the SLAB in facilitating *trans-*splicing, the NUFIP body appears to be located even closer to the *VSG* at the telomere of the active ES, with an average distance of 240 nm compared with 350 nm for the SLAB (Budzak et al., 2022).

Although the function of conserved Cajal body proteins has been investigated in trypanosomes (Barth et al., 2008; Jae et al., 2011), the function of the NUFIP body is unclear. The conserved NUFIP and ZNHIT3 proteins in the *T. brucei* NUFIP body play a role in snRNP assembly in yeast and mammals (Rothe et al., 2014; Bizarro et al., 2015). However, the other three NUFIP body proteins identified do not have known functions, other than that they contain RNA recognition motifs (Budzak et al., 2022). Several NUFIP body components have been shown to co-immunoprecipitate with CRK9, which is involved in RNA modification of the SL RNA, and is essential for *trans*-splicing in *T. brucei* (Badjatia et al., 2016). Cajal bodies frequently associate with highly transcribed loci, including the U1 and U2 snRNA genes in mammalian cells (Smith et al., 1995). In both BF and PF *T. brucei,* the NUFIP body is frequently in close proximity to the SLAB (associating with the highly transcribed SL RNA genes)*,* indicating possible transfer of splicing components (Budzak et al., 2022). A NUFIP body has not yet been identified in other organisms. Therefore, an attractive hypothesis is that the NUFIP body is a novel type of nuclear body similar to the Cajal body, but specifically dedicated to facilitating *trans*-splicing in Kinetoplastid protozoa. The presence of at least one NUFIP body in BF *T. brucei* cells argues that it is an essential structure.

Surprisingly, some NUFIP body proteins were previously identified associating with kinetochore proteins localising to the outer kinetochore during mitosis (Nerusheva et al., 2019; Brusini et al., 2021). Possibly the NUFIP body transiently interacts with the kinetochore during mitosis, indicating that *T. brucei* centromere function requires some aspect of RNA biology. Splicing factors could sometimes associate with kinetochores in mammalian cells, where they have been postulated to have additional secondary functions in the regulation of mitosis (Pellacani et al., 2018; Somma et al., 2020). Further studies are required to characterise the precise function(s) of the NUFIP body in *T. brucei*. However, if proximity of a NUFIP body to the active ES is required for efficient splicing, as most BF *T. brucei* have only one NUFIP body, this could provide an important restriction behind the monoallelic exclusion operating at the active *VSG* expression site.

### Formation of a Nuclear Body Assembly at the Active *VSG* Expression Site

Why are all four nuclear bodies in the proximity of the active ES? Presumably the trypanosome can only keep up with the phenomenal demand for *VSG* mRNA by concentrating both transcription and splicing machineries at the active ES. The dynamic association of the splicing bodies with the active ES could explain how the trypanosome can maintain a *VSG* splicing rate which is 512-fold higher than at a typical Pol II gene (Budzak et al., 2022). If the ESB is considered a Pol I transcription factory, the SLAB, NUFIP and Cajal bodies could be considered mobile splicing component factories, each of which might be optimised to produce different required components. This nuclear body assembly associating at the active ES, could be analogous to a *VSG* mRNA super-factory with mobile subunits. The mobility of these splicing bodies presumably allows them to dynamically provide splicing components throughout the nucleus, collecting in regions of greatest need (Faria et al., 2021). It is unclear why trypanosomes require so many different splicing bodies. Possibly the NUFIP and Cajal bodies are where *T. brucei* performs the final stages of snRNP assembly to generate splicing competent particles. This could create an assembly line, where *VSG* pre-mRNA is generated and processed in a way optimised for maximal expression.

An important question which arises from this model, is how does *VSG* achieve such high expression levels, while *ESAG*s transcribed from the same ES are expressed at significantly lower levels? Possibly *ESAG* mRNAs are selectively degraded at a higher rate than *VSG* mRNA. Knockdown of VEX2 results in upregulation of *ESAG* mRNA from the active ES, suggesting that active RNA degradation suppresses maximal *ESAG* expression (Faria et al., 2019). However, how such selective degradation would occur is unclear. In contrast, *VSG* mRNA is stabilised through RNA binding proteins and RNA modification, which could prevent degradation immediately after transcription (Ridewood et al., 2017; Viegas et al., 2020; Melo do Nascimento et al., 2021). In addition, the SLAB, NUFIP and Cajal bodies appeared to be positioned closer to the ES telomere rather than the promoter, possibly facilitating particularly efficient *VSG* mRNA splicing (Budzak et al., 2022). Collectively, these different mechanisms could be used to achieve higher levels of production of *VSG* compared with *ESAG* mRNA.

This nuclear body assembly could also be important for monoallelic expression of *VSG.* Only one ESB nuclear body assembly is stably present in BF *T. brucei*, where the active ES is transcribed at a very high rate (Budzak et al., 2019; Budzak et al., 2022). Only low levels of transcription are observed immediately downstream of the 14 “silent” ES promoters (Kassem et al., 2014), and these transcripts are not spliced or polyadenylated efficiently (Vanhamme et al., 2000). Silent ESs are not in the same nuclear location as the active ES (Muller et al., 2018; Budzak et al., 2019). If proximity of the active ES to an assembly of transcription and splicing bodies is important for its activation, then exclusion of silent ESs from the ESB nuclear body assembly could maintain them in a silent state. As there is feedback between splicing and transcription elongation at the active ES, increased access of a “silent” ES to pre-mRNA processing machinery, possibly stimulates transcription elongation and therefore its activation. The limited number of nuclear splicing bodies within the BF trypanosome (only one or two of each type) could therefore be a restriction facilitating monoallelic expression of the active ES.

In mammalian cells, nuclear splicing bodies including Cajal bodies are preferentially located at highly transcribed loci, and Cajal body disruption leads to decreased gene expression (Wang et al., 2016). This is also the case for structures called “nuclear speckles”, which are enriched for pre-mRNA splicing factors as well as proteins involved in transcription and post-translational modifications (Galganski et al., 2017; Kim et al., 2020). Analysis of the 3D organisation of the mammalian nucleus showed that genes within transcription “hot zones” are in the proximity of nuclear speckles (Chen et al., 2018). In *T. brucei,* the reorganisation of the nuclear architecture appears to be more extreme, as a collection of up to four nuclear bodies assemble at a single locus. Further work will be required to determine the mechanisms which target these nuclear bodies to the active ES, and whether this targeting is a cause or a consequence of high levels of RNA processing. Although recent discoveries highlight the presence of splicing bodies at the active ES, other components of the pre-mRNA processing machinery involved in polyadenylation or RNA modification may also be concentrated at the active ES. However, this remains to be investigated. It is also unclear how minimally one of the SL RNA gene arrays interacts with the active *VSG* ES.

## Conclusion

In summary, *T. brucei* continues to provide unique molecular solutions for its problems, including how to produce enough VSG (∼10% total protein) for a protective coat, from a single active *VSG* gene. The unusual use of Pol I allows extremely high levels of *VSG* transcription within an ESB. However, extraordinarily high levels of *trans*-splicing are also required at the active *VSG*. This appears to be facilitated by the recruitment of the SL RNA genes, as well as three splicing related nuclear bodies (SLAB, NUFIP and Cajal) to the vicinity of the active ES. These constitute an ES nuclear body assembly functioning as a VSG super-factory. This allows the trypanosome to push the limits of what is possible, to achieve phenomenal levels of expression from a single copy gene.
